# Variation in Leaf Chlorophyll Concentration in Response to Nitrogen Application Across Maize Hybrids in Contrasting Environments

**DOI:** 10.17912/micropub.biology.001115

**Published:** 2024-03-01

**Authors:** Kyle M. Linders, Dipak Santra, James C. Schnable, Brandi Sigmon

**Affiliations:** 1 Department of Agronomy and Horticulture, University of Nebraska–Lincoln, Lincoln, Nebraska, United States; 2 Center for Plant Science Innovation, University of Nebraska-Lincoln, Lincoln, Nebraska, United States; 3 Department of Plant Pathology, University of Nebraska–Lincoln, Lincoln, Nebraska, United States

## Abstract

Leaf chlorophyll concentration was measured for 84 publicly available maize hybrids grown under three nitrogen fertilizer treatments in two contrasting environments in Nebraska. The effect of nitrogen treatment on chlorophyll response was found to be significant (p < 0.05) for both locations. In Scottsbluff, chlorophyll concentrations increased significantly with increasing nitrogen rate, while no significant difference was found between medium and high nitrogen in Lincoln. Within equivalent nitrogen treatments, chlorophyll was more abundant in Lincoln than Scottsbluff for nearly every hybrid. Hybrid response was not consistent between environments, with approximately 11% of variance explained by genotype by environment interaction.

**Figure 1. Chlorophyll concentrations across nitrogen treatments and locations:**
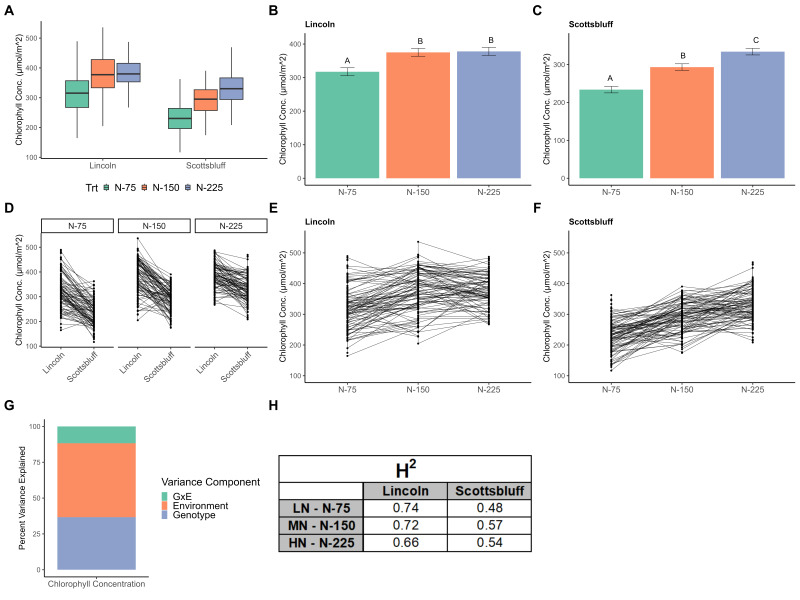
**A)** Boxplot comparing leaf chlorophyll concentration across treatments and locations. **B,C)** Chlorophyll concentration increased significantly with increased nitrogen fertilizer rate in Scottsbluff while HN and MN differed non-significantly in Lincoln. Error bars indicate the least significant difference at 5% significance and letters indicate significance groups. **D,E,F)** Individual hybrids show a plastic response between treatments and locations. **G)** Approximately 11% of the total variance in chlorophyll concentration can be explained by GxE interaction, showing the ability of maize to alter its phenotype in differing environments. **H)** Broad-sense heritabilities show moderately high genetic control of chlorophyll concentration.

## Description

The global population is projected to reach 9.7 billion by 2050, bringing a significant challenge to crop production: to produce more food using less land (United Nations 2022) . Between 1985 and 2005, total global production of cereal crops increased by 29% while harvested area decreased by 3.6% (Foley et al., 2011) . Despite this promising trajectory, further improvements must be made in order to meet the predicted demand. In the past century, a significant portion of yield gain can be attributed to optimizing crop management. The adoption of synthetic nitrogen fertilizer in the United States is estimated to be responsible for a 26% increase in the production of six major non-leguminous crops alone (Stewart et al., 2005) . However, nitrogen fertilizer application has several negative consequences including increased greenhouse gas emissions, pollution of rural groundwater, and runoff into rivers and streams affecting both local and downstream ecosystems. In addition, nitrogen fertilizer is an expensive input in maize production, reducing profit for farmers (Zhang et al., 2015) . Therefore, it is of economic and environmental interest to limit excess nitrogen application and determine precise nitrogen needs to reach full yield potential.

Maize requires sufficient nitrogen throughout the growing season in order to reach its maximum yield potential. Understanding the genetic architecture of maize nitrogen use efficiency will lead to further development of nitrogen efficient, stress tolerant maize hybrids. Significant increases in leaf chlorophyll content were observed at the R1 growth stage with increasing nitrogen fertilizer rates in both irrigated and non-irrigated fields (Széles et al., 2012). Chlorophyll concentration at the R1 growth stage is significantly correlated with both yield and seed protein content (Ványiné et al., 2012). This makes chlorophyll concentration a reliable predictor of plant productivity early in the reproductive stage, allowing for determination of nitrogen fertilizer needs. However, the strength of the correlation of these measurements varied by hybrid and year, indicating a high degree of environmental plasticity (Ványiné et al., 2012).

In order to investigate the phenotypic plasticity of maize leaf chlorophyll concentration between contrasting environments and nitrogen conditions, 84 maize hybrids derived from public sector and expired plant variety patent inbred parents were grown under three nitrogen treatments (Low Nitrogen (LN) – 75 lb/acre, Medium Nitrogen (MN) – 150 lb/acre, High Nitrogen (HN) – 225 lb/acre) with replication in two locations: Lincoln in eastern Nebraska and Scottsbluff in western Nebraska. Scottsbluff is approximately 2,700 ft higher in elevation than Lincoln and receives an average of 13.25 fewer inches precipitation per year. In Scottsbluff, iron deficiency (IDC) was observed across all three nitrogen treatments, leading to interveinal chlorosis. In addition, the Scottsbluff field received supplemental irrigation while Lincoln was rain-fed. These large differences in elevation, precipitation, and soil quality across the state make Nebraska an ideal location for studying plasticity in contrasting environments. Chlorophyll concentration measurements were taken using an Apogee MC-100 Chlorophyll Concentration Meter. This system emits two wavelengths of light to measure the ratio of optical transmission of near-infrared radiation (931 nm) to red light (653 nm). Red light is absorbed by chlorophyll while NIR is reflected, allowing for quantification of leaf chlorophyll content using the chlorophyll content index (CCI). CCI readings are subsequently converted to absolute chlorophyll content in units of µmol/m ^2^ .

Chlorophyll concentration was higher in Lincoln than in Scottsbluff for nearly every hybrid across all three nitrogen treatments. Interestingly, similar concentrations were obtained between Lincoln LN and Scottsbluff HN due to the highly significant location effect ( Fig. 1A ). Within each location, the effect of nitrogen treatment on chlorophyll concentration was statistically significant (p < 0.05). In Lincoln, the LN treatment differed significantly from the MN and HN treatments, with a non-significant (<1%) decrease between HN and MN and an approximately 16% decrease between HN and LN ( Fig. 1B ). In Scottsbluff, all three treatments differed significantly with a 12.2% decrease between HN and MN and a 30% decrease between HN and LN ( Fig. 1C ). Chlorophyll concentration is shown to be moderately genetically controlled with broad-sense heritabilities within each environment (Location/Treatment combination) ranging from 0.48 to 0.74 ( Fig. 1H ).

A mixed model considering genotype (G), environment (N Treatment + Location, E), and genotype by environment (GxE) effects was fit to the chlorophyll concentration data. Approximately 37% of the total variation in chlorophyl concentration can be explained by differences in genotype while ~52% can be attributed to environment. 11% of the total variance in chlorophyll concentration can be explained by GxE, showing a plastic hybrid response to environmental factors ( Fig. 1G ).

## Methods

Fields were planted in two locations at the University of Nebraska-Lincoln’s Havelock Farm in Lincoln (40.852, -96.618), NE and the University of Nebraska Panhandle Research Center in Scottsbluff, NE (N. 41 ^0^ 56’.936, W. 103 ^0^ 42'.164) on 5/20/22 and 5/19/22 respectively. Each field was laid out in a randomized complete block design with three blocks at each location corresponding to three nitrogen application treatments: Low Nitrogen (LN, 75 lbs/acre), Medium Nitrogen (MN, 150 lbs/acre), and High Nitrogen (HN, 225 lbs/acre). Eighty-four hybrids were replicated twice in each block for a total of approximately 170 plots block and 510 plots per field. Each plot consisted of four 17.5 ft long rows of a single hybrid planted at 30-inch row spacing. The Scottsbluff field received supplemental moisture through a center pivot irrigation system while the Lincoln field was non-irrigated. Data collection took place in a single day on 7/26 (67 DAS) in Lincoln and 8/1 (74 DAS) in Scottsbluff. The fields were at a similar developmental stage with an estimated 40-60% of plots at or past the R1 growth stage.

Chlorophyll concentration was measured using an Apogee MC-100 Chlorophyll Concentration Meter on two representative plants from the middle of each plot. Measurements were collected on the third fully developed leaf from the top of the plant. On each left, three measurements were taken starting at the base and moving towards the tip avoiding the midrib to account for in-leaf variability (Ge et al., 2019) . All six measurements from each plot were averaged to obtain a final plot chlorophyl concentration in µmol/m ^2^ .

Statistical analyses were conducted in R v.4.1.2 (R Core Team 2021) . The meta-package *tidyverse* (Wickhamet al., 2019) was utilized for data processing and visualization. Data distributions were visually checked for outliers and non-biologically meaningful outliers were removed. Between 3 and 12 plots from each location/treatment combination were omitted from the analysis due to outlier determination or missing data due to plot damage. To compare the impact of the treatment effect across hybrids and between locations, mixed models were fit to the chlorophyll concentration data using the *lmer* function within the package *lme4* (Bates et al., 2015) . A model was fit to chlorophyll measurements from both the Lincoln and Scottsbluff fields containing nitrogen treatment as a fixed effect and genotype as a random effect. Fisher’s least significant difference (LSD) was used to obtain p-values for treatment effects with values below 0.05 considered significant. A third model was fit to the combined dataset which designated each treatment/location combination as a separate environment. The model contained environment as a fixed effect and genotype, and well as genotype by environment interaction (GxE) as random effects. This allows determination of the proportion of total variance explained by these three effects.

To calculate broad-sense heritability, simplified models were fit to each environment to obtain genetic and residual variances. Broad-sense heritabilities were estimated using the following equation:

H^{2} = \frac{\sigma _{g}^{2}}{\sigma _{g}^{2} + \frac{\sigma _{e}^{2}}{n}}

With \sigma _{g}^{2} corresponding to genetic variance, \sigma _{e}^{2} to residual variance, and n to the number of replicates.
